# The impact of a ‘milking the COW’ campaign in a regional hospital in Singapore

**DOI:** 10.1186/s13756-021-00948-1

**Published:** 2021-05-22

**Authors:** Surinder Kaur M. S. Pada, Poh Lishi, Kim Sim Ng, Sarathamani Rethenam, Lilibeth Silagan Alenton, Poh Ling Chee, Wilma Guo, Yin Maw Hsann, Carmen Wan Rong Cheng, Chiou Horng Ong, Ratnayake Lasantha, Douglas Chan, Paul Anantharajah Tambyah

**Affiliations:** 1 Division of Infectious Diseases, Department of Medicine, Ng Teng Fong General Hospital, 1 Jurong East Street 21, Singapore, 609606 Singapore; 2 Infection Control, Department of Nursing, Ng Teng Fong General Hospital, Singapore, Singapore; 3 Department of Epidemiology, Ng Teng Fong General Hospital, Singapore, Singapore; 4 Department of Laboratory Medicine, Ng Teng Fong General Hospital, Singapore, Singapore; 5 Infectious Diseases Translational Research Programme, Department of Medicine, Yong Loo Lin School of Medicine, National University of Singapore, Singapore, 119228 Singapore

## Abstract

**Background:**

Computerisation of various processes in hospitals and reliance on electronic devices raises the concern of contamination of these devices from the patient environment. We undertook this study to determine if an attached hand hygiene device that unlocks the screen of a computer on wheels (COW) on usage can be effective in decreasing the microbiological burden on computer keyboards.

**Methods:**

An electronic hand sanitizer was integrated onto the COW. A prospective cohort study with a crossover design involving 2 control and 2 intervention wards was used. The study end point was the number of colony forming units found on the keyboards. Bacteria were classified into 4 main groups; pathogenic, skin flora, from the environment or those thought to be commensals in healthy individuals. We then used a mixed effects model for the statistical analysis to determine if there were any differences before and after the intervention.

**Results:**

Thirty-nine keyboards were swabbed at baseline, day 7 and 14, with 234 keyboards cultured, colony forming units (CFUs) counted and organisms isolated. By mixed model analysis, the difference of mean bacteria count between intervention and control for week 1 was 32.74 (− 32.74, CI − 94.29 to 28.75, *p* = 0.29), for week 2 by 155.86 (− 155.86, CI − 227.45 to − 83.53, *p* < 0.0001), and after the 2-week period by 157.04 (− 157.04, CI − 231.53 to − 82.67, *p* < 0.0001). In the sub-analysis, there were significant differences of pathogenic bacteria counts for the Intervention as compared to the Control in contrast with commensal counts.

**Conclusion:**

A hand hygiene device attached to a COW may be effective in decreasing the microbiological burden on computer keyboards.

**Supplementary Information:**

The online version contains supplementary material available at 10.1186/s13756-021-00948-1.

## Background

Electronic medical records and the computerisation of various processes in modern day hospitals have made us increasingly reliant on computers and other electronic devices. Contamination of these devices from the patient environment is a significant concern. Many investigators have documented contamination of computer keyboards with pathogenic micro-organisms. While some have formally assessed effectiveness of cleaning, none have looked specifically at the impact of hand hygiene at the point of use of computer keyboards [1–6]. A summary of these studies are included in Additional file 1. We undertook to determine if an attached hand hygiene device that unlocks the screen of a computer on wheels (COW) only on usage can be effective in decreasing the microbiological burden on computer keyboards. We aimed to study the effect of this innovative approach to hand hygiene on the level of contamination of keyboards on our COWs.

A proof of concept study on a smaller scale (4 COWS) was undertaken in 2015 when we occupied a 350 bedded hospital while awaiting the move to a new hospital campus with 700 beds. The results of POC study encouraged us to conduct a larger study to determine the actual impact of usage of such a device.

## Methods

**Fig. 1:**
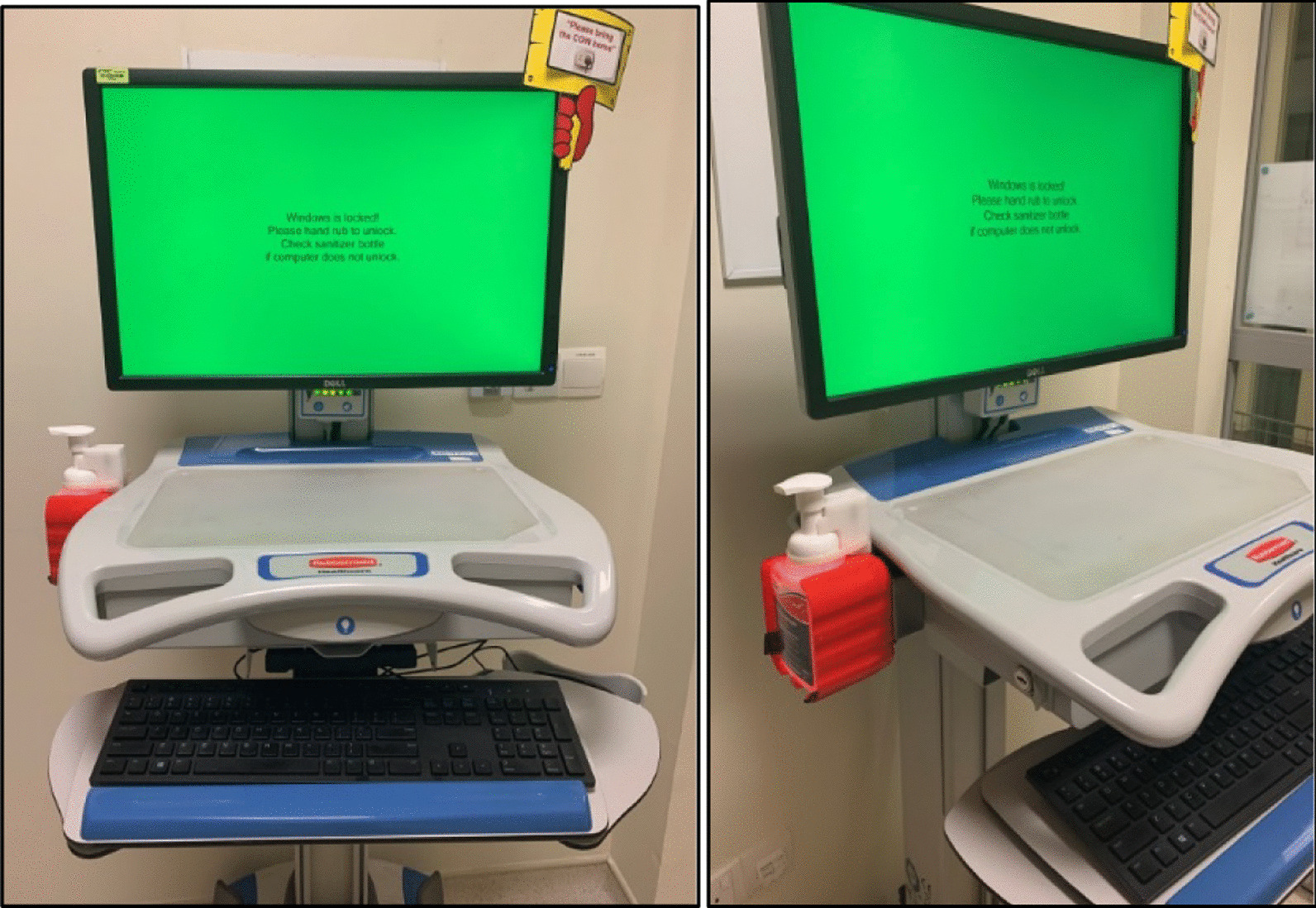
Set up of the COW with the hand hygiene devise attached

Software was developed by members of the team that linked the hand hygiene device to the computer. The log in screen of these COWS could only be activated by using the attached hand sanitizer (see Fig. 1). In addition an LED light projected through the hand hygiene dispenser flashed red when the COW was logged out and would turn green only on successful activation by use of the hand hygiene dispenser to signal that logging in could proceed. We set a time-out duration of 120 s after activation before a subsequent default locked screen would appear if a COW was left idle. This time-out duration could be adjusted if the unit staff overall felt that it was insufficient, there was no minimum or maximum limit that could be set.

We conducted a prospective cohort study with a crossover design involving 2 control wards (one general medical and one surgical) and 2 intervention wards (also one general medical and one surgical). The first phase of the study was completed within 6 weeks, from 31/10/2017 to 5/12/2017. Hand hygiene devices were installed onto 2 interventions wards for 2 weeks before crossing over to the 2 control wards after a one week wash out period. In the first 2-week block, there were 19 COWs in the intervention arm, and 20 COWS in the control arm. After crossing over, there were 20 COWs in the intervention arm and 19 in the control arm.

We followed the standardised cleaning schedule with routine cleaning of the COWs from 1500 to 1630 h daily. This ensured that the intervention and control wards followed a consistent cleaning regime. The chemical compositions of the hospital disinfectant used were as followed: 100 g of agent solutions contains 0.26 g Quaternary ammonium compounds, benzyl-C-12-16-alkydimethyl, chlorides; 0.26 g Didecyldimethylammonium chloride, 0.26 g Quaternary ammonium compounds, C12-14-alkyl [(ethylphenyl) methyl] dimethyl, chlorides with a recommended contact time of 1 min.

The study end point was the number of colony forming units found on the keyboards by our pre-determined microbiological surveillance methods. We developed a protocol to swab the keyboards as detailed in Table 1.

**Table 1:** Summary of the planned steps for microbial sampling

Week	2 (intervention) wards	2 (control) wards	Total samples
Day D-1 10 am–1 pm	Swab all COWs keyboards (baseline)	Swab all COWs keyboards (baseline)	39 swabs
Day 0 12 pm–4 pm	Attach Hand hygiene dispenser on all COWs	No Hand hygiene dispenser attached	
Day 7 10 am–1 pm	Swab all COWs Keyboards	Swab all COWs Keyboards	39 swabs
Day 14 10 am–1 pm	Swab all COWs keyboards	Swab all COWs keyboards	39 swabs
*Wash out period for a week*			
Day 0 10 am–1 pm	Repeat swabs for all COWs keyboards	Repeat swabs for all COWs keyboards	39 swabs
Day 0 12 pm–4 pm	Attach Hand hygiene dispenser on all COWs	No Hand hygiene dispenser attachment	
Day 7 10 am–12 pm	Swab all COWs Keyboards	Swab all COWs Keyboards	39 swabs
Day 14 10 am–12 pm	Swab all COWs keyboards	Swab all COWs keyboards	39 swabs

The keyboards were divided into two halves from the centre letters Y, H & N. One flocked swab was used to swab the surface on the left starting from keyboard letters Y, H, & N and another flocked swab was used to swab the surface of the keyboard right side. Care was taken to swab the surface of the keys only. This process took approximately 20 s. Laboratory staff were on site to ensure adequate sampling and for immediate transferring of the swabs to the microbiology laboratory for processing. To ensure uniformity, the same person was responsible for sampling all the keyboards.

### Microbiological methods

Samples were collected with flocked swabs and transported promptly to the microbiology laboratory in 1 mL liquid Amies media (Copan Italia SpA, Brescia, Italy). To facilitate growth of the bacteria of interest, an inoculum of 0.1 mL of the media was plated by Kiestra™ InoqulA™ (Becton Dickinson, Franklin Lakes, NJ, USA) directly on tryptic soy agar with 5% sheep blood (TSA 5% sheep blood) (Becton, Dickinson and Company, Sparks, MD) and MacConkey agar (Thermo Fisher Scientific, Melaka, Malaysia). Plates were streaked with InoqulA standard pattern (Stardard_PatternSet_highspeed_2011_12_01) to resemble spread-plating to enumerate viable bacterial/ fungal colonies [7]. After 18 to 24 h’ incubation at 35 °C ± 2 °C in ambient air, colonies with different morphology were identified using MALDI-TOF (MALDI-TOF MS; Bruker Daltonics, Germany), details of which have been published elsewhere [8]. For bacteria identified as *Enterobacterales*, *Enterococcus faecium* or *faecalis* and *Staphylococcus aureus* further workup was performed according to EUCAST guidelines to rule out Carbapenemase-Producing E*nterobacterales* (CPE), Vancomycin Resistant *Enterococcus* (VRE) and Methicillin-resistant *Staphylococcus aureus* (MRSA) [9]. Suspected mould colonies were propagated on sabouraud dextrose agar (SAB) (Becton, Dickinson and Company, Sparks, MD) and identification was performed based on macroscopic and microscopic features [10]. Plates that showed no growth of organism after the initial incubation were incubated for an additional 18–24 h.

Viable count calculation was done for individual organism type with 1 colony observed on the agar plate equivalent to 10 colony forming unit (CFU)/mL.

The bacteria were grouped according to those that were deemed pathogenic, skin flora, from the environment or those thought to be commensals in healthy individuals [11–14]. The details of the classification can be found in the Additional file 2: Table S1.

### Staff survey on ease of use

A survey on ease of use and overall impressions from staff was undertaken. Data were collected in 2 separate 1 week blocks, whilst the device was in use on the interventional wards. There were 122 staff surveyed which included 93 nurses, 7 doctors and 22 others which included therapists, patient care assistants, students etc. The questions asked can be found in Additional file 3: Table S2.

### Statistical analysis

Linear mixed effect models were used to compare the mean bacteria counts between the intervention arm and the control arm in the study periods, accounting for the random variability in wards and COWs. Due to different ward location for the COWs and the repeated samples collected from the COWs, random effects were included to take account for the non-independence in data. The dependent variable was bacteria count, the random effects were ward and COW and the fixed effect was study arm (intervention or control). The estimated mean differences and their 95% confidence intervals were reported. Differences were considered significant for a *p* value < 0.05. The outcome measure was a decrease in the microbiological burden on computer keyboards with the use of the Hand Hygiene device. The analysis was performed by statistical software R 3.4.2

### Ethics

This was a quality initiative and had been approved by the Board of Trustees of the hospital. Informed consent from health care workers was therefor not obtained.

## Results

A total of 39 keyboards were swabbed at baseline, day 7 and day 14. In total, 234 keyboards were cultured to identify the bacteria counts and organisms isolated. The main analysis showed evidence of difference on bacteria counts for the Intervention as compared to the Control for week 2 and the 2-week period taken as a whole but not week one. By the mixed model, the difference of mean bacteria count for week 1 was 32.74 (− 32.74 CI − 94.29 to 28.75, *p* = 0.29), The mean bacteria count after week 2 for the Intervention was lower than the Control by 155.86 (− 155.86, CI − 227.45 to − 83.53, *p* < 0.0001), and the mean bacteria count after 2-week period for the Intervention was lower than the Control by 157.04 (− 157.04, CI − 231.53 to − 82.67, *p* < 0.0001).

In the sub-analysis, there were significant differences of pathogenic bacteria counts for the Intervention as compared to the Control in contrast with commensal counts. By the mixed model, the mean of pathogenic bacteria counts for the Intervention as compared to the Control reduced in week 1, week 2 and the total 2-week period were by 4.01 (CI (− 7.61 to − 0.42), *p* < 0.05), 19.39 (CI − 30.30 to − 8.36), *p* < 0.001) and 18.85 (CI (− 30.03 to − 7.67), *p* < 0.001). For environmental bacteria, the mean bacteria counts for the Intervention as compared to the Control after week 1 increased by 17.42 (*p* < 0.01). This may be because the large value “1100” in the day 7 results, which inflated the results after week 1. For skin flora, the results were similar to the overall results with significant reductions of bacteria counts for the Intervention as compared to the Control for week 2 and 2-week period but not week 1. There were few commensal bacteria in most analyses. Of note, in terms of Multi-drug resistant organisms, only MRSA was detected in the control arm. No VRE or CRE were detected in this study. A summary of the results can be found in Table 2. The details of the raw CFU counts can be found in the Additional file 4: Table S3.

**Table 2:** Main Analysis and Sub Analysis study

Models/variables	Difference in mean bacteria count	95% confidence interval	*P* value
*Main analysis*			
Group Week 1	− 32.74	(− 94.29, 28.75)	0.29 NS
Group Week 2	− 155.86	(− 227.45, − 83.53)	< 0.0001
Group Total	− 157.04	(− 231.53, − 82.67)	< 0.0001
*Subgroup analysis*			
Pathogenic organisms			
Week 1	− 4.01	(− 7.61, − 0.42)	< 0.05
Week 2	− 19.39	(− 30.30, − 8.36)	< 0.001
Total	− 18.85	(− 30.03, − 7.67)	< 0.001
Environmental organisms			
Week 1	17.43	(4.62, 30.21)	< 0.05
Week 2	− 1.58	(-3.74, 0.58)	0.15 NS
Total	− 1.671915	(-3.77, 0.42)	0.12 NS
Skin flora			
Week 1	− 38.72	(− 97.55, 19.93)	0.1929 NS
Week 2	− 138.34	(− 208.68, − 66.91)	< 0.001
Total	− 135.01	(− 207.22, − 62.95)	< 0.001
Commensal organisms			
Week 1	0.52	(− 4.22, 5.25)	0.83 NS
Week 2	− 0.76	(− 1.73, 0.22)	0.12 NS
Total	− 0.77	(− 1.75, 0.21)	0.12 NS
Non-pathogenic organisms			
Week 1	− 24.19	(− 85.91, 37.53)	0.43 NS
Week 2	− 137.66	(− 208.75, − 66.57)	< 0.001
Total	− 136.49	(− 209.40, − 63.58)	< 0.001

**Fig. 2:**
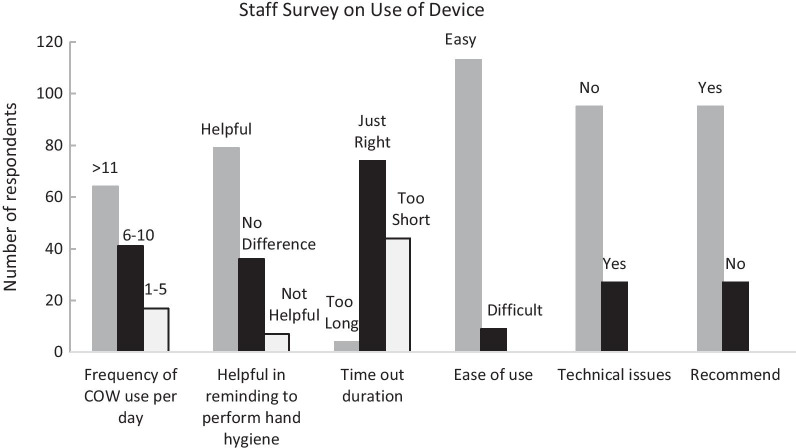
Results of survey on use of device

Survey results on the use of the device on the interventional wards are summarised in Fig. 2 below.

## Discussion

This is a first study of its kind to show a reduction in microbial contamination of keyboards by using a hand hygiene dispenser at the point of use of the COW. It was a cross over study and included both medical and surgical wards so should be generalizable.

We had also developed a standardised method of sampling the keyboards with the same personnel deployed for collection of the swabs to reduce variability. We were also able to collect a large number of swabs, totalling nearly 240 over the study period for which we speciated all colonies identified using Matrix-Assisted Laser Desorption Ionization Time-of-Flight Mass Spectrometry (MALDI TOF). As such we were able to make meaningful subgroup analyses.

Of note, the overall study found a marked reduction in the mean bacteria count after the 2-week period for the Intervention versus the Control ward of 157.04 (− 157.04, CI − 231.53, − 82.67) colonies despite identical high touch cleaning schedules suggesting that this reduction of the bioburden was truly due to the intervention.

Subgroup analyses also confirmed that this was most marked for pathogenic organisms, in contrast with environmental organisms, which we assume would be removed by high touch surface cleaning and not typically found on the hands of health care workers. This suggests that the prompts from the device had a significant impact on hand hygiene as evidenced by reduction in bacteria most likely carried on the hands of healthcare workers.

Although in the main analysis, significant differences were only seen in week 2 and as a total, this could be explained by behavioural change requiring time to take effect. It could be reasonable to expect that it might have taken 2 weeks for the intervention to produce benefits. Alternatively, as seen in the sub-group analysis, those classified as pathogens decreased in both weeks 1 and 2, whereas environmental pathogens had increased in week 1 before decreasing in week 2. The results of the main analysis hence, probably allude to the fact that environmental pathogens may be harder to eradicate and are not directly related to hand hygiene.

Whilst a number of studies have shown that contaminated surfaces of equipment used in healthcare settings may act as fomites and transmit pathogens, and that adequate cleaning may help to reduce the level of contamination, none have addressed the additive effect of hand hygiene at the point of use [1–6]. Whilst Lu et al. reported lower contamination rates of their computer keyboards attributed to better hand hygiene compliance in the absence of a cleaning programme, this was not specifically quantified [3]. The staff survey was also helpful in understanding the practicality of this device. Of note, 93% of staff found the device easy to use. Although the time out duration of 120 s was deemed just right by 61%, our software was flexible enough to allow us to increase or decrease the timing based on the experience of our users, so we did not view this as a major issue. Although 22% of our staff experienced technical issues, these were mostly resolved quickly. With familiarity of setting up the software and hardware gained through this study, as well as with the anticipated development of a more robust hardware in the next phase of the project, we hope to have addressed the remaining issues raised. Ultimately, the majority of our staff surveyed (78%) affirmed that they would recommend deployment of the device throughout the hospital.

This intervention is also likely to drive behaviour change since it is an added reminder of “Moment 5: After touching patient surroundings” according to the WHO moments of Hand Hygiene and highlights the fact that healthcare equipment are fomites that can cause disease transmission. Due to the short duration of the intervention, we were not able to corroborate this with hand hygiene data from our institution.

One of the major limitations of our study is that a reduction in CFU does not necessarily translate to reduction in infections. There are no data in the published literature that can attest to a clear cut causal relationship between the reduction in CFU count and decrease in hospital acquired infections. Studies of environmental cleaning have provided some evidence that a reduction in the bioburden of pathogens in the environment, particularly high touch surface areas, has resulted in reductions in healthcare associated infections [15, 16]. Numerous reports have shown that improving hand hygiene rates have also reduced hospital acquired infections [17–20]. It is thus probably reasonable to assume that we can expect some reduction in hospital acquired infections with optimal use of our device.

The relatively short period of the intervention of 2 weeks, although sufficient to study the effects on the organisms found on keyboards, was not long enough to allow us to collect data for surveillance of patient infection and colonization with pathogens such as MRSA and C difficile. If we have the resources to do a further, larger and longer study, it could provide further corroborative evidence to promote the widespread implementation of such a device.

Another limitation of the study was the that, due to the nature of the intervention, blinding of the team carrying out the sampling could not be done. However, we did try to limit any study bias by having a team consisting of Infection Control nurses as well as laboratory staff carry out the sampling independently of the ward teams. We further used this same team throughout the study period to ensure consistency in sampling.

It is also possible that the results could have been due to less staff using the COWs in the interventional wards since the device was clearly visible and required the user to undergo an extra step in what was already a very busy ward environment. We do not, however, think that this was a major issue. We use an electronic medical record system throughout our facility. The only means of documentation when at the patient’s bedside is through the use of the COW. Alternative desktop computers are only found at the nurses’ station and substation and are not as numerous as COWs. Also, our survey results showed that 86% of staff used the device more than 6 times per day meaning that most people did not change their behaviour to avoid using the COW. In addition, the finding of skin flora in the interventional wards also provided some corroborative evidence that the COWs were still being used and that our results could still be interpreted positively.

## Conclusion

A hand hygiene device attached to a computer on wheels (COW) that unlocks the screen on usage may be effective in decreasing the microbiological burden on computer keyboards. Computerised devices act as reservoirs for pathogenic microorganisms and potentially promote transfer of the pathogens to patients. Hand hygiene as an intervention prior to usage may be a relatively simple but worthwhile intervention in preventing nosocomial infection.

## Supplementary Information

**Additional file 1**: Summary of studies investigating the levels of contamination on electronic devices

**Additional file 2**: **Table S1.** Organisms categorised as pathogenic, environmental, skin flora and commensals

**Additional file 2**: **Table S2.** Questions asked for staff survey on ease of use

**Additional file 4**: **Table S3.** Colony forming units and pathogens isolated from individual computer keyboards over the study period

## Data Availability

All data generated or analysed during this study are included in this published article and its supplementary information files.
