# Supplementary material for: The impact of a ‘milking the COW’ campaign in a regional hospital in Singapore
Source: Antimicrob Resist Infect Control. 2021 May 22;10:81. doi: 10.1186/s13756-021-00948-1 (PMC8141142; doi:10.1186/s13756-021-00948-1)
Supplement: Supplementary file 1 — Additional file 1: Summary of studies investigating the levels of contamination on electronic devices [file 13756_2021_948_MOESM1_ESM.docx]

Supplementary Materials 1:

Summary of studies investigating levels of contamination on electronic devices

| Study | Setting | Relevant Results | Recommendation |
| --- | --- | --- | --- |
| Rutala et al.^1^ | Assessed the effectiveness of 6 different disinfectants. | Sampled 25 computers and found: n(%)  OSSA 1 (4), ORSA 1 (4),  VSE 3 (12), CoNS 25 (100)  Diphtheroids 20 (80),  *Micrococcus* species 18 (72)  *Bacillus* species 16 (64),  NF-GNR 9 (36),  Propionibacteria 7 (28),  Alpha streptococci 6 (21),  Viridans streptococci 2 (8),  *Aspergillus niger* 5 (20)  *Aspergillus flavus* 1 (4) | Microbial contamination of keyboards is prevalent and that keyboards may be successfully decontaminated  with disinfectants. |
| Messina et al.  ^2^ | -Cross-over study  -Italian teaching hospital  Evaluated microbial contamination of keyboards, telephone handsets and stethoscopes before and after  cleaning  Differences in contamination between hospital units and between stethoscopes and keyboards plus handsets analysed | The percentage of positive  samples was higher on computer keyboards, followed by telephone handsets and stethoscopes.  27 computer keyboards evaluated:  E coli: 11/27 (40.7%)  Coliforms: 21/27 (77.8%)  Enterococci: 4/27 (14.8%)  Staphylococcus: 25/27 (92.6%)  MRSA: 6/27 (22.2%)  Moulds: 20/27 (74.1%) | Healthcare professionals should disinfect stethoscopes and other possible sources of bacterial  healthcare-associated infections. |
| Lu et al.^3^ | A 1600 bed medical center in Taiwan , 47 wards and 282 computers.  Cross sectional surveillance,  investigated the association of  methicillin-resistant *Staphylococcus aureus* (MRSA), *Pseudomonas aeruginosa* and *Acinetobacter*  *baumannii*, from ward computer keyboards, mice and  from clinical isolates | 17.4% (49/282) contamination rate of these computer devices by *S. aureus*, *Acinetobacter* spp. or *Pseudomonas* spp. The contamination rates of MRSA and *A. baumannii* in the ward computers were 1.1% and 4.3%, respectively | With good hand hygiene compliance,  no necessity of routine culture surveillance in non-outbreak  situation. |
| Moore et al.^4^ | Study to investigate the distribution of hospital pathogens within general and critical care ward environments.  Prospective 4-month microbiological survey | Regardless of the ward, surfaces closest to the patient, specifically those associated with the bed (side rails, bed control, and call button), were the most heavily contaminated. In the ICU, bacteria were most likely to be on surfaces that were regularly touched by healthcare workers (e.g., telephones and computer keyboards). | Different ward types should be treated as separate  environments, and cleaning protocols should be adjusted accordingly. |
| Hartmann et al. ^5^ | A 14 bed surgical intensive  care unit  Examine the microbial contamination of computer user interfaces with pathogenic microorganisms, compared with other fomites. | Enterococcus 12/222 (5.5%)  Staph aureus 3/222 (1.2%)  GNR 2/222 (0.9%) | The colonization rate for  computer keyboard is greater than that of other user  interfaces and may be additional  reservoirs for the transmission of microorganisms |
| Srikanth et al. ^6^ | Study to measure, compare and characterize the aerobic microorganisms in computer keyboards of hospital and non-hospital settings. | Growth was seen in all 80 samples (40 from each setting). *Staphylococcus aureus* was isolated from both settings, hospital: 6 MRSA (15%), 11 MSSA (27.5%)  non-hospital: 4 MRSA (10%), 9 MSSA (22.5%)  Gram-negative bacilli were isolated more frequently from hospital settings (33%). | Isolation of microorganisms from “high-touch” surfaces such as computer keyboards is indicative  of the need for awareness on cleaning of such surfaces and adequate hand hygiene. |
