# Supplementary material for: The impact of a ‘milking the COW’ campaign in a regional hospital in Singapore
Source: Antimicrob Resist Infect Control. 2021 May 22;10:81. doi: 10.1186/s13756-021-00948-1 (PMC8141142; doi:10.1186/s13756-021-00948-1)
Supplement: Supplementary file 2 — Additional file 2: Table S1. Organisms categorised as pathogenic, environmental, skin flora and commensals [file 13756_2021_948_MOESM2_ESM.docx]

| **Pathogenic organisms** |
| --- |
| *Acinetobacter baumannii, Candida parapsilosis, Enterococcus faecium, Klebsiella pneumoniae, Methicillin resistant Staphylococcus aureus (MRSA), Methicillin susceptible Staphylococcus aureus (MSSA), Staphylococcus lugdunensis, Streptococcus dysgalactiae* |
| **Environmental organisms** |
| *Acinetobacter species: Acinetobacter calcoaceticus, Acinetobacter schindleri, Acinetobacter towneri, Acinetobacter ursingii, Arthrobacter sulfonivorans, Arthrobacter woluwensis, Brevundimonas diminuta,*  *Bacillus species: Bacillus cereus, Bacillus circulans, Bacillus flexus, Bacillus gibsonii, Bacillus infantis, Bacillus licheniformis, Bacillus luciferensis, Bacillus marisflavi, Bacillus megaterium, Bacillus mycoides, Bacillus oshimensis, Bacillus pumilus, Bacillus sonorensis, Bacillus subtilis group, Bacillus thuringiensis, Curtobacterium albidum, Chryseobacterium indologenes,*  *Gram negative rods: Lysinibacillus species, Microbacterium species*  *Mould: non-sporulating, sporulating, Aspergillus niger, Aspergillus niger complex, Curvularia species, Penicillum spp, suspected Zygomycetes*  *Pseudomonas species: Pseudomonas alcaligenes, Pseudomonas mendocina, Pseudomonas oryzihabitans, Pseudomonas putida*  *Other: Brevibacillus species, Paenibacillus species, Paenibacillus jamilae, Pantoea dispersa, Pseudoxanthomonas species, Streptomyces badius, Roseomonas gilardii, Roseomonas mucosa* |
| **Skin flora** |
| *Aerococcus* spec*ies: Aerococcus viridans, Corynebacterium species: Corynebacterium glutamicum, Corynebacterium propinquum, Corynebacterium xerosis, Dermabacter hominis, Gram positive rods, Gram positive cocci, Kocuria species, Kocuria kristinae, Kocuria palustris, Kytococcus species, Micrococcus species, Micrococcus luteus, Corynebacterium species, Staphylococcus species, Staphylococcus auricularis, Staphylococcus capitis, Staphylococcus caprae, Staphylococcus cohnii, Staphylococcus condiment, Staphyloccocus epidermidis, Staphylococcus haemolyticus, Staphylococcus hominis, Staphylococcus kloosii, Staphylococcus pettenkoferi, Staphylococcus saprophyticus, Staphyloccocus warneri, Staphylococcus xylosus* |
| **Commensal organisms** |
| *Brevibacterium casei, Brevibacterium paucivorans, Cellulosimicrobium cellulans, Leclercia adecarboxylata, Moraxella species, Moraxella osloensis, Neisseria species, Pantoea agglomerans group, Rothia mucilaginosa, Streptococcus mitis group, Streptococcus oralis, Streptococcus sanguinis, Streptococcus species, Trichosporon species* |

Table 1: Organisms categorised as Pathogenic, Environmental, Skin flora and Commensals.
