# Supplementary material for: The impact of a ‘milking the COW’ campaign in a regional hospital in Singapore
Source: Antimicrob Resist Infect Control. 2021 May 22;10:81. doi: 10.1186/s13756-021-00948-1 (PMC8141142; doi:10.1186/s13756-021-00948-1)
Supplement: Supplementary file 3 — Additional file 2: Table S2. Questions asked for staff survey on ease of use [file 13756_2021_948_MOESM3_ESM.docx]

| **1)How often did you use the COW hand hygiene device?** |
| --- |
| **2)Does the COW hand hygiene device help to remind you to perform hand hygiene?** |
| **3)Is the COW hand hygiene device easy to use?** |
| **4)What do you think about the time-out duration (120 seconds) for the COW?** |
| **5)Have you encountered any technical issues with the usage of the device?** |
| **6)Do you recommend this device for future COWs?** |

Table 2: Questions asked for staff survey on ease of use
