# Supplementary material for: The impact of a ‘milking the COW’ campaign in a regional hospital in Singapore
Source: Antimicrob Resist Infect Control. 2021 May 22;10:81. doi: 10.1186/s13756-021-00948-1 (PMC8141142; doi:10.1186/s13756-021-00948-1)
Supplement: Supplementary file 4 — Additional file 4: Table S3. Colony forming units and pathogens isolated from individual computer keyboards over the study period [file 13756_2021_948_MOESM4_ESM.docx]

| **Organism type:**  **Control ward** | **Colony count (CFU/mL)** | | |
| --- | --- | --- | --- |
|  | **21/11/2017**  **Day 0** | **28/11/2017**  **Day 7** | **5/12/2017**  **Day14** |
| **5SC1**  Pathogenic  Environmental  Skin flora  Commensals | 0  0  0  0 | 0  10  130  0 | 40  0  190  0 |
| **Total** | **0** | **140** | **230** |
| **5SC2**  Pathogenic  Environmental  Skin flora  Commensals | 0  10  0  0 | 0  10  410  0 | 0  0  160  0 |
| **Total** | **10** | **420** | **160** |
| **5SC3**  Pathogenic  Environmental  Skin flora  Commensals | 0  0  10  0 | 0  10  170  0 | 0  0  100  0 |
| **Total** | **10** | **180** | **100** |
| **5SC4**  Pathogenic  Environmental  Skin flora  Commensals | 0  0  0  0 | 0  0  80  0 | 0  0  100  0 |
| **Total** | **0** | **80** | **100** |
| **5SC5**  Pathogenic  Environmental  Skin flora  Commensals | 0  10  20  0 | 10  0  70  30 | 0  0  10  0 |
| **Total** | **30** | **110** | **10** |
| **5SC6**  Pathogenic  Environmental  Skin flora  Commensals | 0  0  0  0 | 0  10  40  0 | 0  10  130  0 |
| **Total** | **0** | **50** | **140** |
| **5SC7**  Pathogenic  Environmental  Skin flora  Commensals | 0  10  250  0 | 0  10  230  0 | 200  0  500  0 |
| **Total** | **260** | **240** | **700** |
| **5SC8**  Pathogenic  Environmental  Skin flora  Commensals | 0  0  0  0 | 10  0  40  0 | 0  0  0  10 |
| **Total** | **0** | **50** | **10** |
| **5SC9**  Pathogenic  Environmental  Skin flora  Commensals | 0  0  0  0 | 0  20  60  0 | 0  0  50  0 |
| **Total** | **0** | **80** | **50** |
| **5SC10**  Pathogenic  Environmental  Skin flora  Commensals | 0  10  10  0 | 0  20  0  0 | 0  30  10  0 |
| **Total** | **20** | **20** | **40** |
| **5SC11**  Pathogenic  Environmental  Skin flora  Commensals | 0  0  10  0 | 0  0  10  0 | 0  10  330  10 |
| **Total** | **10** | **10** | **350** |
| **8SC1**  Pathogenic  Environmental  Skin flora  Commensals | 0  30  40  0 | 0  10  10  0 | 0  0  20  0 |
| **Total** | **70** | **20** | **20** |
| **8SC2**  Pathogenic  Environmental  Skin flora  Commensals | 0  10  0  0 | 0  10  0  0 | 0  0  20  0 |
| **Total** | **10** | **10** | **20** |
| **8SC3**  Pathogenic  Environmental  Skin flora  Commensals | 0  0  0  0 | 0  0  10  0 | 0  0  70  0 |
| **Total** | **0** | **10** | **70** |
| **8SC4**  Pathogenic  Environmental  Skin flora  Commensals | 0  0  0  0 | 0  10  30  0 | 0  0  170  0 |
| **Total** | **0** | **40** | **170** |
| **8SC5**  Pathogenic  Environmental  Skin flora  Commensals | 0  0  0  0 | 0  0  30  0 | 0  0  20  0 |
| **Total** | **0** | **30** | **20** |
| **8SC6**  Pathogenic  Environmental  Skin flora  Commensals | 0  0  60  0 | 0  10  20  0 | 0  0  >1000  0 |
| **Total** | **60** | **30** | **>1000** |
| **8SC7**  Pathogenic  Environmental  Skin flora  Commensals | 0  0  0  0 | 0  0  20  0 | 0  0  0  0 |
| **Total** | **0** | **20** | **0** |
| **8SC8**  Pathogenic  Environmental  Skin flora  Commensals | 0  10  50  0 | 0  10  20  0 | 0  0  >1000  0 |
| **Total** | **60** | **30** | **>1000** |

| **Organism type:**  **Intervention ward** | **Colony count (CFU/mL)** | | |
| --- | --- | --- | --- |
|  | **30/10/2017**  **Baseline** | **7/11/2017**  **Day 7** | **14/11/2017**  **Day14** |
| **5SC1**  Pathogenic  Environmental  Skin flora  Commensals | 0  0  70  0 | 0  0  50  0 | 10  10  0  0 |
| **Total** | **70** | **50** | **20** |
| **5SC2**  Pathogenic  Environmental  Skin flora  Commensals | 0  0  10  0 | 0  10  10  0 | 0  0  20  0 |
| **Total** | **10** | **20** | **20** |
| **5SC3**  Pathogenic  Environmental  Skin flora  Commensals | 0  0  20  0 | 20  0  20  50 | 0  0  0  10 |
| **Total** | **20** | **90** | **10** |
| **5SC4**  Pathogenic  Environmental  Skin flora  Commensals | 0  20  0  0 | 0  10  40  0 | 0  0  80  0 |
| **Total** | **20** | **50** | **80** |
| **5SC5**  Pathogenic  Environmental  Skin flora  Commensals | 0  0  0  0 | 0  0  40  0 | 0  0  0  0 |
| **Total** | **0** | **40** | **0** |
| **5SC6**  Pathogenic  Environmental  Skin flora  Commensals | 0  0  10  0 | 0  10  20  0 | 0  0  0  0 |
| **Total** | **10** | **30** | **0** |
| **5SC7**  Pathogenic  Environmental  Skin flora  Commensals | 0  0  0  0 | 0  0  10  0 | 0  0  10  0 |
| **Total** | **0** | **10** | **10** |
| **5SC8**  Pathogenic  Environmental  Skin flora  Commensals | 0  10  0  0 | 0  0  10  0 | 0  0  0  0 |
| **Total** | **10** | **10** | **0** |
| **5SC9**  Pathogenic  Environmental  Skin flora  Commensals | 0  0  10  0 | 0  0  10  10 | 0  0  0  0 |
| **Total** | **10** | **10** | **0** |
| **5SC10**  Pathogenic  Environmental  Skin flora  Commensals | 0  0  0  0 | 0  0  0  0 | 0  20  70  0 |
| **Total** | **0** | **0** | **90** |
| **5SC11**  Pathogenic  Environmental  Skin flora  Commensals | 0  0  0  0 | 0  0  10  0 | 0  0  0  0 |
| **Total** | **0** | **10** | **0** |
| **8SC1**  Pathogenic  Environmental  Skin flora  Commensals | 0  10  30  0 | 0  0  70  0 | 0  0  0  0 |
| **Total** | **40** | **70** | **0** |
| **8SC2**  Pathogenic  Environmental  Skin flora  Commensals | 0  0  0  0 | 0  0  130  20 | 0  0  0  0 |
| **Total** | **0** | **150** | **0** |
| **8SC3**  Pathogenic  Environmental  Skin flora  Commensals | 0  0  10  0 | 0  0  0  0 | 0  0  0  0 |
| **Total** | **10** | **0** | **0** |
| **8SC4**  Pathogenic  Environmental  Skin flora  Commensals | 0  0  30  0 | 0  0  60  0 | 30  0  0  0 |
| **Total** | **30** | **60** | **30** |
| **8SC5**  Pathogenic  Environmental  Skin flora  Commensals | 0  0  0  0 | 0  0  0  0 | 0  0  0  0 |
| **Total** | **0** | **0** | **0** |
| **8SC6**  Pathogenic  Environmental  Skin flora  Commensals | 0  0  10  0 | 0  0  50  0 | 0  0  0  0 |
| **Total** | **10** | **50** | **0** |
| **8SC7**  Pathogenic  Environmental  Skin flora  Commensals | 0  0  20  0 | 0  10  0  0 | 0  0  0  0 |
| **Total** | **20** | **0** | **0** |
| **8SC8**  Pathogenic  Environmental  Skin flora  Commensals | 0  0  >1000  0 | 0  40  10  0 | 0  0  10  0 |
| **Total** | **>1000** | **50** | **10** |

| **Organism type:**  **Control ward** | **Colony count (CFU/mL)** | | |
| --- | --- | --- | --- |
|  | **30/10/2017**  **Baseline** | **7/11/2017**  **Day 7** | **14/11/2017**  **Day14** |
| **9SC1**  Pathogenic  Environmental  Skin flora  Commensals | 0  0  0  0 | 90  0  330  80 | 0  0  10  0 |
| **Total** | **0** | **500** | **10** |
| **9SC2**  Pathogenic  Environmental  Skin flora  Commensals | 0  0  10  0 | 0  10  0  0 | 0  10  210  0 |
| **Total** | **10** | **10** | **210** |
| **9SC3**  Pathogenic  Environmental  Skin flora  Commensals | 0  10  20  0 | 0  0  120  40 | 0  10  0  0 |
| **Total** | **30** | **160** | **10** |
| **9SC4**  Pathogenic  Environmental  Skin flora  Commensals | 0  10  280  0 | 0  10  120  0 | 220  0  10  0 |
| **Total** | **290** | **130** | **230** |
| **9SC5**  Pathogenic  Environmental  Skin flora  Commensals | 0  0  10  0 | 0  0  200  0 | 0  0  60  0 |
| **Total** | **10** | **200** | **60** |
| **9SC6**  Pathogenic  Environmental  Skin flora  Commensals | 0  30  10  0 | 0  20  50  0 | 120  50  120  0 |
| **Total** | **40** | **70** | **290** |
| **9SC7**  Pathogenic  Environmental  Skin flora  Commensals | 0  0  480  0 | 0  10  80  40 | 80  0  150  0 |
| **Total** | **480** | **130** | **230** |
| **9SC8**  Pathogenic  Environmental  Skin flora  Commensals | 0  0  40  10 | 0  0  300  0 | 0  0  0  0 |
| **Total** | **50** | **300** | **0** |
| **9SC9**  Pathogenic  Environmental  Skin flora  Commensals | 0  50  20  0 | 40  0  90  0 | 0  0  >1000  0 |
| **Total** | **70** | **130** | **>1000** |
| **9SC10**  Pathogenic  Environmental  Skin flora  Commensals | 0  0  40  0 | 0  10  300  10 | 10  0  10  0 |
| **Total** | **40** | **320** | **20** |
| **9SC11**  Pathogenic  Environmental  Skin flora  Commensals | 0  30  100  0 | 0  10  50  0 | 40  0  230  0 |
| **Total** | **130** | **60** | **270** |
| **16SC1**  Pathogenic  Environmental  Skin flora  Commensals | 0  10  220  20 | 0  0  50  20 | 0  0  >1000  0 |
| **Total** | **250** | **70** | **>1000** |
| **16SC2**  Pathogenic  Environmental  Skin flora  Commensals | 0  0  50  10 | 0  0  110  0 | 0  10  10  0 |
| **Total** | **60** | **110** | **20** |
| **16SC3**  Pathogenic  Environmental  Skin flora  Commensals | 0  30  340  0 | 0  30  80  0 | 40  0  >1000  0 |
| **Total** | **370** | **110** | **>1000** |
| **16SC4**  Pathogenic  Environmental  Skin flora  Commensals | 0  0  120  0 | 10  10  210  0 | 0  0  10  0 |
| **Total** | **120** | **240** | **10** |
| **16SC5**  Pathogenic  Environmental  Skin flora  Commensals | 0  10  150  0 | 0  10  50  0 | 10  0  10  0 |
| **Total** | **160** | **60** | **20** |
| **16SC6**  Pathogenic  Environmental  Skin flora  Commensals | 0  0  0  0 | 20  10  280  0 | 0  0  10  0 |
| **Total** | **0** | **310** | **10** |
| **16SC7**  Pathogenic  Environmental  Skin flora  Commensals | 0  210  0  110 | 0  0  150  10 | 0  0  30  0 |
| **Total** | **320** | **160** | **30** |
| **16SC8**  Pathogenic  Environmental  Skin flora  Commensals | 0  10  530  0 | 10  0  200  0 | 0  0  10  0 |
| **Total** | **540** | **210** | **20** |
| **16SC9**  Pathogenic  Environmental  Skin flora  Commensals | 0  150  310  0 | 0  10  >1000  10 | 0  0  0  0 |
| **Total** | **460** | **>1020** | **0** |

| **Organism type:**  **Intervention ward** | **Colony count (CFU/mL)** | | |
| --- | --- | --- | --- |
|  | **21/11/17**  **Day 0** | **28/11/2017**  **Day 7** | **5/12/2017**  **Day14** |
| **9SC1**  Pathogenic  Environmental  Skin flora  Commensals | 70  0  820  0 | 0  260  210  0 | 0  0  10  0 |
| **Total** | **890** | **370** | **10** |
| **9SC2**  Pathogenic  Environmental  Skin flora  Commensals | 0  0  70  0 | 0  0  80  0 | 0  0  0  0 |
| **Total** | **70** | **80** | **0** |
| **9SC3**  Pathogenic  Environmental  Skin flora  Commensals | 0  1100  10  0 | 0  90  80  0 | 0  10  30  0 |
| **Total** | **1110** | **170** | **40** |
| **9SC4**  Pathogenic  Environmental  Skin flora  Commensals | 0  0  0  0 | 10  10  150  20 | 0  0  60  0 |
| **Total** | **0** | **190** | **60** |
| **9SC5**  Pathogenic  Environmental  Skin flora  Commensals | 120  0  110  0 | 0  20  90  40 | 0  10  80  0 |
| **Total** | **230** | **150** | **90** |
| **9SC6**  Pathogenic  Environmental  Skin flora  Commensals | 0  0  >1000  0 | 0  10  180  0 | 0  0  60  0 |
| **Total** | **>1000** | **190** | **60** |
| **9SC7**  Pathogenic  Environmental  Skin flora  Commensals | 0  60  1520  0 | 0  0  70  0 | 0  10  10  0 |
| **Total** | **1580** | **70** | **20** |
| **9SC8**  Pathogenic  Environmental  Skin flora  Commensals | 0  0  40  0 | 0  10  100  0 | 0  0  10  0 |
| **Total** | **40** | **110** | **10** |
| **9SC9**  Pathogenic  Environmental  Skin flora  Commensals | 0  30  20  0 | 0  0  100  50 | 0  0  20  0 |
| **Total** | **50** | **150** | **20** |
| **9SC10**  Pathogenic  Environmental  Skin flora  Commensals | 0  20  10  0 | 0  20  180  0 | 0  10  0  10 |
| **Total** | **30** | **200** | **20** |
| **9SC11**  Pathogenic  Environmental  Skin flora  Commensals | 10  0  90  0 | 0  20  180  0 | 0  10  0  10 |
| **Total** | **100** | **200** | **20** |
| **16SC1**  Pathogenic  Environmental  Skin flora  Commensals | 10  0  220  0 | 0  0  250  0 | 0  0  10  0 |
| **Total** | **230** | **250** | **10** |
| **16SC2**  Pathogenic  Environmental  Skin flora  Commensals | 0  0  30  0 | 0  0  350  70 | 0  0  30  0 |
| **Total** | **30** | **420** | **30** |
| **16SC3**  Pathogenic  Environmental  Skin flora  Commensals | 0  0  340  120 | 0  270  190  10 | 0  0  70  0 |
| **Total** | **460** | **470** | **70** |
| **16SC4**  Pathogenic  Environmental  Skin flora  Commensals | 0  0  0  0 | 0  10  0  0 | 0  0  40  0 |
| **Total** | **0** | **40** | **40** |
| **16SC5**  Pathogenic  Environmental  Skin flora  Commensals | 10  10  80  0 | 0  50  80  0 | 0  0  10  0 |
| **Total** | **100** | **130** | **10** |
| **16SC6**  Pathogenic  Environmental  Skin flora  Commensals | 0  10  20  0 | 0  40  260  30 | 0  0  >870  0 |
| **Total** | **30** | **330** | **>870** |
| **16SC7**  Pathogenic  Environmental  Skin flora  Commensals | 230  0  90  0 | 0  0  >1000  0 | 0  0  190  0 |
| **Total** | **320** | **>1000** | **190** |
| **16SC8**  Pathogenic  Environmental  Skin flora  Commensals | 300  30  60  0 | 0  10  240  20 | 40  0  20  0 |
| **Total** | **390** | **270** | **60** |
| **16SC9**  Pathogenic  Environmental  Skin flora  Commensals | 0  70  230  10 | 0  10  160  0 | 0  0  80  0 |
| **Total** | **310** | **170** | **80** |

Supplementary material Table 3: Colony Forming units and pathogens isolated from individual computer keyboards over the study period.
